# Rationale and protocol of a trial for prevention of diabetic atherosclerosis by using antiplatelet drugs: study of Diabetic Atherosclerosis Prevention by Cilostazol (DAPC study)

**DOI:** 10.1186/1475-2840-5-16

**Published:** 2006-08-22

**Authors:** Yoshimitsu Yamasaki, Young-Seol Kim, Ryuzo Kawamori

**Affiliations:** 1 Department of Internal Medicine and Therapeutics, Osaka University Faculty of Medicine, 2-2 Yamadaoka, Suita, Osaka 565-0871, Japan; 2 Department of Internal Medicine, Kyunghee University College of Medicine, 1 Hoeki-dong, Dongdaemoon-ku, Seoul, 130-702, Korea; 3 Department of Medicine, Metabolism & Endocrinology, Juntendo University School of Medicine, 2-1-1 Hongo, Bunkyo-ku, Tokyo 113-8421, Japan

## Abstract

**Background:**

Secondary treatment of arteriosclerosis may be applicable for the primary prevention of atherosclerosis in diabetic patients. This prospective, 2-year follow-up study was designed to determine the efficacy and safety of antiplatelet therapy in the prevention of atherosclerosis of diabetic subjects.

**Methods:**

Patients with type 2 diabetes and arteriosclerosis obliterans from the Eastern Asian countries were registered online and randomly assigned either to the aspirin group (81–100 mg/day) or the cilostazol group (100–200 mg/day) in this international, 2-year, prospective follow-up interventional study.

**Results:**

The primary study endpoint was changes in right and left maximum intima-media thickness of the common carotid artery. Secondary endpoints include changes in right and left maximum intima-media thickness of the internal carotid artery; semiquantitative evaluation of cerebral infarction by magnetic resonance imaging; cardiovascular events including sudden death, stroke, transient cerebral ischemic attacks, acute myocardial infarction, angina, and progression of arteriosclerosis obliterans; overall death; withdrawal; and change in ankle-brachial pressure index.

**Conclusion:**

This is the first study to use an online system that was developed in Asian countries for pooling data from an international clinical trial. These findings are expected to help in the prevention of diabetic atherosclerosis and subsequent cardiovascular and cerebrovascular disease.

## Background

Westernization of lifestyle has led to an explosive increase in the number of diabetic patients and an increase in diabetic atherosclerosis in Eastern Asian countries [1,2]. This is of great concern for the future of patients and health systems in East Asia, and is a problem that requires immediate and careful evaluation. With this in mind a cooperative, multinational research group in Japan, Korea, China, and Phillippine has been formed to investigate the potential for antiplatelet medication to prevent the occurrence and progression of diabetic atherosclerosis in Asian patients.

The rationale for the study is based upon the finding that the primary incidence rate of coronary artery disease (CAD) in diabetic patients is similar to the secondary incidence rate of CAD in nondiabetic patients. This finding suggests that secondary treatment of atherosclerosis is applicable to the primary prevention of arteriosclerosis in diabetic patients. Antiplatelet drugs are widely reported to be effective in preventing the recurrence of atherosclerosis. In one study [3], type 2 diabetic patients with early-stage carotid atherosclerosis who were given aspirin showed a slight progression of intima-media thickness (IMT) of the carotid artery (0.033 ± 0.010 mm/year). In another study [4], similar patients given cilostazol showed negligible change in IMT (0.00 ± 0.16 mm/3 years). However, the primary preventive and secondary therapeutic effects of these two drugs have not been sufficiently investigated in the Asian diabetic patient population. We therefore initiated an international, 2-year prospective follow-up interventional study to clarify the efficacy and usefulness of aspirin and cilostazol in the primary prevention and secondary treatment of diabetic atherosclerosis in Asian patients. Assessment will be performed by chronologically observing the intima-media thickness (IMT) of the carotid artery, which is used as a surrogate endpoint of atherosclerosis, and by analyzing the occurrence and progression of large-vessel complications in patients with type 2 diabetes and mild atherosclerosis [5,6].

### Study design and protocol

The DAPC study has been registered on the University Hospital Medical Information NetworkClinical Trials Registry (UMIN-CTR) which is a non-profit organization in Japan and meets the requirements of the International Committee of Medical Journal Editors (ICMJE). Patients enrolled in the study will have been dignosed with type 2 diabetes, aged between 40 to 85 years and with clinical findings suggestive of arteriosclerosis obliterans (ASO) (Table 1, Table 2). In the study ASO will be defined as detection of the following findings in either of the patient's lower limbs: an ABI (ankle brachial pressure index) of <1.0 and a weakened or bilaterally different pulsation of the popliteal artery or dorsal artery of the foot; or clinical signs and symptoms suggestive of ASO.

**Table 1:** Enrollment Criteria

***Inclusion criteria:***
1) Type 2 diabetes mellitus
2) Age between 40 to 85 years
3) Clinical findings suggestive of arteriosclerosis obliterans (ASO)
Note 1: Definition of ASO: Patient who has the following lesions in either of the lower limbs
• ABI (ankle brachial pressure index) < 1.0
• A weakened or bilaterally different pulsation of popliteal artery or dorsal artery of foot
• clinical signs and symptoms suggestive of ASO
***Exclusion criteria:***
1) Type 1 diabetes mellitus or secondary diabetes mellitus
2) Age less than 40 or greater than 85 years
3) Severe ASO rated as Fontaine IIb or over
4) Cerebrovascular disorders rated as grade 4 or greater on the Modified Rankin Scale
5) Medical history of angina or myocardial infarction
6) Severe hepatic dysfunction (liver cirrhosis) or renal dysfunction (serum creatinine ≥ 1.5 mg/dL)
7) Congestive heart failure
8) Severe hematological abnormalities
9) Diagnosis of homologous familial hypercholesterolemia
10) Drug allergies or medical history of hypersensitivity to the investigational drugs
11) Pregnant or lactating women, or women who wish to become pregnant
12) Others whom the investigator judges inappropriate as subjects for this study

**Table 2:** Study Outline

**Target number of patients: **408 patients	
**Study Design: **Randomization	Aspirin group (81–100 mg/day; Group A) Cilostazol group (100–200 mg/day; Group C)
**Follow-up: **2-year prospective follow-up study	
**Primary endpoints:**	
IMT*: Variation of right and left max IMT-CCA and mean IMT-CCA	
**Secondary endpoints:**	
IMT*: Variation of right and left max IMT-ICA	

*Variation is measured by the IMT Evaluation Committee by using a diagnostic software application called IntimaScope^® ^while the allocations are blinded.

The following exclusion criteria were set: those with type 1 or secondary diabetes, severe ASO (greater than a Fontaine IIb category) or grade 4 or higher cerebral infarction on the Modified Rankin Scale, and medical history of angina or myocardial infarction. In addition, patients with a range of concomitant comorbidities including severe hepatic or renal dysfunction, congestive heart failure, bleeding disorders, or familial hypercholesterolemia, those with allergy or hypersensitivity to the investigational drugs, pregnant or lactating women, or women wishing to become pregnant, were excluded. Written informed consent has been obtained from all patients, and the study has been reviewed by the ethics review board of the all institutes participated in this study if the institute had the ethics review board.

Registration of patients was preformed at the administration office of the Diabetic Atherosclerosis Prevention by Cilostazol (DAPC) Study via the Internet, and once enrolled, subjects were randomly assigned to the aspirin group (81–100 mg/day; group A) or the cilostazol group (100–200 mg/day; group C). Patients who were already receiving antiplatelet drugs were assigned without a washout period. The study period will be 2 years after the registration of subjects (registration period: November 2004 to March 2006; full study duration: December 2004 to March 2008).

The primary study endpoint is the change in right and left maximum IMT of the common carotid artery (CCA) and mean IMT-CCA change. Secondary endpoints are the change in right and left maximum IMT of the internal carotid artery; semiquantitative evaluation of cerebral infarction by MRI measurement; cardiovascular events including sudden death, occurrence or recurrence of stroke, transient cerebral ischemic attacks, acute myocardial infarction, angina, and progression of ASO; overall death; withdrawal; and change in ABI.

Investigations will be performed at the start of the study, after 1 year, at the conclusion of the study, and at the time of any discontinuations or changes in dosage. Any adverse reactions will also be assessed.

Statistical analysis using changes in IMT was first undertaken to determine the number of patients required to identify a significant difference between the two groups. As a result, at least 408 patients were calculated to be required to obtain a significant difference (*p *< 0.05) between the two groups. Therefore, the target number of patients was 400 for the 1-year registration period. Statistical analysis will be performed using the *t*-test to compare the change in maximum IMT, the log-rank test for incidence of events and mortality rate, and the Kaplan-Meier method to plot survival curves. Ratios of events between the 2 groups and confidence intervals are to be determined by Cox regression. A 1-way ANOVA will be used to compare ABI. The administrative office of the DAPC Study will analyze these data.

## Discussion

The aim of this study is to clarify the efficacy and usefulness of two different antiplatelet drugs, aspirin and cilostazol, in the prevention of occurrence or progression of atherosclerosis in patients with type 2 diabetes. Antiplatelet drugs such as aspirin have been shown to be effective in preventing the recurrence of atherosclerosis in these patients. However, effectiveness of aspirin on diabetic patients is still controversial. Kodama and colleagues [3] have reported that both aspirin and ticlopidine significantly reduced, but did not halt, progression of increase in IMT in patients with type 2 diabetes and early-stage carotid atherosclerosis. Recently, the Primary Prevention Project (PPP) Trial showed that a significant reduction of the total cardiovascular events was observed in nondiabetic subjects but no significant reduction was observed in diabetic subjects [7]. In contrast, Shinoda-Tagawa and colleagues [4] showed that similar patients given cilostazol showed a negligible change in IMT over a 3-year follow-up period. Cilostazol is also shown to be effective in reducing occurrence of cerebral infarction in diabetic subjects [4]. We feel that the therapeutic effects resulting from the differing mechanisms of action of aspirin and cilostazol have not been sufficiently investigated in this patient population.

The results of this study are expected to assist in the development of appropriate drug therapies for patients with type 2 diabetes and atherosclerosis. This is a very important objective because the incidence of this common complication of diabetes is rising at a staggering rate among many Asian countries. It is expected that health systems in Asia will be severely stretched in the near future by trying to manage patients with diabetic complications, and the economic and social costs to the individuals and countries will be large. The design of this study is ground breaking in that enrollment was via the Internet and all results will be pooled by using a unique online patient registry system. Not only are all of the mathematical and demographic data to be held on this database, but also all of the images taken from the MRI assessment of cerebral changes and alterations in IMT. This is the first such online system to be developed in Asian countries for pooling data from an international clinical trial. Results will be available rapidly, and these findings are expected to provide clinical data that will help in the prevention of diabetic atherosclerosis and subsequent cardiovascular and cerebrovascular disease. Results from this important clinical trial are planned to be published in 2008.

## Authors' contributions

Yoshimitsu Yamasaki and Young-Seol Kim both directed the study, and Ryuzo Kawamori is the head representative for this study. All authors conceived the study, and participated in its design. Yoshimitsu Yamasaki drafted the manuscript, and all authors approved the final manuscript.

**Appendix:** DAPC Study Participating Institutions and Investigators

**Investigator**	**Institution**
**Japan**	
Goto T	Department of Internal Medicine, Japanese Red Cross Akita Hospital, Akita
Imano A	Department of Internal Medicine, Osaka Kouseinenkin Hospital, Osaka, Japan
Jinnouchi H	Department of Internal Medicine, Jinnouchi Hospital, Kumamoto, Japan
Kanazawa A	Department of Internal Medicine, Division of Metabolism and Endocrinology, Juntendo University School of Medicine, Tokyo, Japan
Kasayama S	Department of Molecular Medicine, Osaka University Graduate School of Medicine, Osaka, Japan
Kosugi K	Department of Internal Medicine, Osaka Police Hospital, Osaka, Japan
Matsuoka T	Kurashiki Life Style Disease Center, Okayama, Japan
Nakato T	Department of Internal Medicine, Okayama Saiseikai General Hospital, Okayama, Japan
Ono T	Department of Diabetes and Endocrine, Iwaki Kyoritsu Hospital, Fukushima, Japan
Ota M	Department of Comprehensive Medical Care, Ota Nishinouchi Hospital, Fukushima
Seino H	Center of Diabetes, Ota Nishinouchi Hospital, Fukushima, Japan
Sumiya T	Department of Endocrine and Diabetes, NTT West Osaka Hospital, Osaka, Japan
Yagi M	Department of Endocrinology and Metabolism, Belland General Hospital, Osaka, Japan
Yamasaki Y*	Department of Endocrine and Metabolic Internal Medicine, Osaka University Graduate School of Medicine, Osaka, Japan
**Korea**	
Chung MY	Department of Internal Medicine, Chonnam National University College of Medicine, Gwangju, Korea
Kim YS*	Department of Internal Medicine, Kyunghee University College of Medicine, Seoul, Korea
Lee HC	Department of Internal Medicine, Yonsei University College of Medicine, Seoul, Korea
Soon KH	Department of Internal Medicine, Keimyung University College of Medicine, Daegu, Korea
Yoon KH	Department of Internal Medicine, The Catholic University of Korea, Seoul, Korea
**Philippines**	
Ganzon MS	Department of Vascular Medicine, Heart Institute, St. Luke’s Medical Center, Manila, Philippines
**China**	
Guo X	Department of Endocrinology, Peking University First Hospital, Beijing, China

*Study director
